# Where should “Humans” be in “One Health”? Lessons from COVID-19 for One Health

**DOI:** 10.1186/s12992-024-01026-y

**Published:** 2024-03-25

**Authors:** Zhaohui Su, Dean McDonnell, Ali Cheshmehzangi, Barry L. Bentley, Sabina Šegalo, Claudimar Pereira da Veiga, Yu-Tao Xiang

**Affiliations:** 1 School of Public Health, Institute for Human Rights, Southeast University, Nanjing, 210009 China; 2 Department of Humanities, South East Technological University, Carlow, R93 V960 Ireland; 3 Center of Innovation for Education and Research (CIER), Qingdao City University, Qingdao, China; 4 Network for Education and Research On Peace and Sustainability, Hiroshima University, Hiroshima, 739-8530 Japan; 5 Cardiff School of Technologies, Cardiff Metropolitan University, Cardiff, UK; 6 Collaboration for the Advancement of Sustainable Medical Innovation, University College London, London, UK; 7 Faculty of Health Studies, University of Sarajevo, 71000 Sarajevo, Bosnia and Herzegovina; 8 Fundação Dom Cabral - FDC, Av. Princesa Diana, 760 Alphaville, Lagoa Dos Ingleses, Nova Lima, MG 34018-006 Brazil; 9 Unit of Psychiatry, Department of Public Health and Medicinal Administration; Institute of Translational Medicine, Faculty of Health Sciences; Centre for Cognitive and Brain Sciences; Institute of Advanced Studies in Humanities and Social Sciences, University of Macau, Taipa, Macao SAR China

**Keywords:** One Health, Human Health, Human-Centred Connected Health, Health Policy, Ethics

## Abstract

The culling of animals that are infected, or suspected to be infected, with COVID-19 has fuelled outcry. What might have contributed to the ongoing debates and discussions about animal rights protection amid global health crises is the lack of a unified understanding and internationally agreed-upon definition of “One Health”. The term One Health is often utilised to describe the imperative to protect the health of humans, animals, and plants, along with the overarching ecosystem in an increasingly connected and globalized world. However, to date, there is a dearth of research on how to balance public health decisions that could impact all key stakeholders under the umbrella of One Health, particularly in contexts where human suffering has been immense. To shed light on the issue, this paper discusses whether One Health means “human-centred connected health” in a largely human-dominated planet, particularly amid crises like COVID-19. The insights of this study could help policymakers make more informed decisions that could effectively and efficiently protect human health while balancing the health and well-being of the rest of the inhabitants of our shared planet Earth.

## Introduction

The world is interconnected. The interaction between humans and animal health, as seen in zoonotic infectious diseases, has been a crucial force that has shaped the course of current and previous pandemics [1]. While differing voices are present, numerous species of animals, ranging from rats to bats, have been widely implicated as carriers of deadly pathogens that have caused or substantially contributed to devastating pandemics throughout human history, ranging from the Black Death, Ebola outbreaks, to the COVID-19 (coronavirus 2019) pandemic [2–5]. Despite the importance and inevitability of human-animal interactions [2], the progress of contemporary understanding of “One Health” has been disappointingly lukewarm and lacklustre. While the concept gained traction amid the severe acute respiratory syndrome (SARS) pandemic starting in 2003, meaningful debates and discussions on One Health are still lacking in academia and beyond. One of the earliest sets of One Health principles, for instance, was developed in the Wildlife Conservation Society’s symposium in 2004 by an international group of experts from fields such as public, veterinary, and environmental health [6]. The principles, which are often referred to as the “Manhattan Principles”, set twelve priorities that have the potential to help society better combat threats to human and animal health [6].

Ranging from calling for greater emphasis on understanding the link between humans, animals, and various diseases, to more impactful investment in education to increase awareness of the interconnectedness of all living species, essentially the “Manhattan Principles” formed the modern concept of “One Health, One World” [6]. Even without drawing on the “butterfly effect” rationale, the importance of acknowledging, understanding, and tackling the interconnectedness of the health and well-being of all species living on Earth can be hardly overemphasized. Take the current pandemic for instance. While much remains to be uncovered, it is highly possible that SARS-CoV-2 (severe acute respiratory syndrome coronavirus 2), the virus that caused COVID-19, might be zoonotic in nature—a virus transmitted from animals to humans, possibly as a result of increasingly frequent human-animal interactions [7–9]. Second, the growing body of evidence on COVID-19 transmissions between humans and animals further underscores the need to investigate and protect public and global health through the lens of One Health. Across the pandemic continuum, insights from research labs and real-world settings both confirm that the list of animals that could either contract COVID-19 or have the ability to infect humans with the virus has been growing [10–13].

In addition to wild animals (e.g., bats, deer, pumas, and lions) and farm animals (e.g., mink), SARS-CoV-2 has also been found in common pets like dogs and cats [13–16]. Growing evidence further shows that the susceptibility of dogs and cats to COVID-19 could range from 0.79% to 40% [16–20]. Considering the prevalence of COVID-19 in domestic animals, along with their close proximity to humans, domestic animals like dogs and cats could pose considerable harm to humans or the overall pandemic control efforts without proper measures. Furthermore, what makes the virus transmissions between humans and animals particularly worrisome also centres on the fact that these transmissions often indicate potential virus mutations—the virus has evolved to the extent that it has the potential to transmit between species, rather than among humans, which could, in turn, lead to unknown consequences (e.g., reservoirs for secondary zoonotic infections) [21]. In addition, the role of these interactions in the course of the pandemic is also evidenced in the suspected animal-to-human transmissions of SARS-CoV-2.

### One health controversies

In early January 2022, health experts in Hong Kong linked COVID-19 infections that first occurred in a pet store to imported hamsters [22]. Later tests confirmed the assumption—10% of the hamsters tested were positive for COVID-19 infections [23]. As a result, the authorities responded by culling over 2,000 hamsters and other small animals, citing the possibility of virus mutations with the potential to further exacerbate the pandemic, including outbreaks caused by animal-human transmissions of the virus [23]. A global outcry ensued over the city’s decision to euthanize these animals en masse, with people questioning whether the decision to cull these animals—many of which may not have been infected with the virus—was necessary [24]. At the time, it was still unclear with regard to hamsters’ transmissibility across COVID-19 variants. What is clear, though, is that preliminary data suggested that at least 50 people were infected with the Delta variant that could be traced back to contact with these hamsters [12]. What is also clear is that the public’s  response was particularly impassioned, and emotionally charged, which further deepened the already divided and fragmented narratives surrounding COVID-19 with regard to pandemic policies (e.g., masking, lockdowns, vaccination) and the general discourse and language around the pandemic (e.g., using biased and discriminatory terms like “Wuhan Virus” or “Chinese Virus” to refer to SARS-CoV-2) [25–28].

One potential contributing factor to the heated discussions around animal rights during the pandemic, particularly from the academic and policy perspective, was the lack of a unified understanding of One Health [29]. To date, there has yet to be a rigorous and internationally accepted definition of the concept, partly because current definitions often do not clearly reflect the competing interests between different One Health sectors as highlighted by COVID-19 and beyond. For instance, one definition provided by the United States Centres for Disease Control and Prevention refers to One Health as “a collaborative, multisectoral, and transdisciplinary approach—working at the local, regional, national, and global levels—with the goal of achieving optimal health outcomes recognizing the interconnection between people, animals, plants, and their shared environment” [30]. In a similar vein, a definition that was given by the World Health Organization’s One Health High-Level Expert Panel (OHHLEP) framed One Health as “an integrated, unifying approach that aims to sustainably balance and optimize the health of people, animals, and ecosystems” [31].

While these definitions could shed some light on the concept of One Health, they nonetheless fail to provide the level of detail necessary to guide health experts’ decision-making in crucial situations, such as how policies should be developed when the interests of humans, animals, and plants are not in tandem or harmony with one another. In other words, in situations where animals become an imminent or almost inevitable threat to human health, as we have seen in cases across the COVID-19 pandemic, which principles should government and health experts utilize in determining which health policies to develop and deploy? Essentially, the current understanding of the One Health concept could hardly address questions such as: Should humans, animals, and plants be considered equal in rights and importance under the concept of One Health, even in times when human health is under imminent and consequential threats like COVID-19? To shed light on the issue, this analysis aims to investigate whether One Health should prioritise human health in a world where many populations face existential challenges like hunger and poor health, particularly in the post-COVID era.

### Where should human health stand under one health?

The idea of putting people’s health first over that of the floras and faunas, if not the universe as a whole, is often in direct contrast to many implicit or stated principles of One Health (please see Table 1). For instance, the first fundamental principle of One Health proposed by OHHLEP is “equity between sectors and disciplines”, with “sectors” referring to humans, animals, and the environment [31]. Overall, of the seven principles established and endorsed by OHHLEP—equity, inclusivity, equal access, parity, socioecological equilibrium, stewardship, and transdisciplinarity, at least three (equity, equilibrium, and stewardship) require humans put an equal, if not a more rigorous, emphasis on the health of the animals and the wider environment [31]. Yet even within the context of these seven One Health principles, there are notable discrepancies, such as the disconnect between the stated goal of protecting and respecting the health of all sectors, the proposed pathways, or the lack thereof, and the reality. For instance, the second set of principles is centring on “sociopolitical and multicultural parity” where “all people are equal and deserve equal rights and opportunities” [31].

**Table 1:** Example One Health Definitions

Source	One Health is Defined As
Davis et al. (2017)	“the intersection and integration of knowledge regarding humans, animals, and the environment” [32]
Hillier et al. (2021)	“the use of multidisciplinary approaches in the implementation of policy design and public health interventions” [33]
One Health High Level Expert Panel (2022)	“an integrated, unifying approach that aims to sustainably balance and optimize the health of people, animals, and ecosystems” [31]
Peterson et al. (2021)	“an approach to achieve better health outcomes for humans, animals, and the environment through collaborative and interdisciplinary efforts” [34]
Sutradhar & Zaman (2021)	“approaching issues of global health by looking at the areas of human, animal, and environmental health, and their intersections” [35]
United States Centers for Disease Control and Prevention	“a collaborative, multisectoral, and transdisciplinary approach — working at the local, regional, national, and global levels — with the goal of achieving optimal health outcomes recognizing the interconnection between persons, animals, plants, and their shared environment” [36]
World Health Organization (2017)	“an integrated, unifying approach to balance and optimize the health of people, animals and the environment. It is particularly important to prevent, predict, detect, and respond to global health threats such as the COVID-19 pandemic” [37]

This principle, in light of the raging and oftentimes blatant gender, racial and ethnic disparities that are present in societies large and small [25, 38], especially post-COVID, might be best described as distantly ideal that is almost impossible to address in the near future. The absence of a detailed pathway as to how the principle of parity might be achieved in the increasingly fragmented post-pandemic world further highlights the issue. This disconnect between the stated principle, the contrasting reality, and the lack of practical pathways, then, raises the question: In a world where people have yet to learn to respect one another for their oftentimes added and artificial “labels” even against continuous interventions and movements, is it even possible to expect society at large to prioritize the allocation of respect and resources for largely alien-looking, foreign-tongued, and possibly disease-carrying non-human inhabitants of the Earth? Existing evidence suggests that there is a notable prioritisation of human health among One Health professionals. Via interviewing six professionals in the human domain and seven in the veterinary domain, for instance, the findings show that, while in principle, a holistic view toward One Health is widely held among the participants, in practice, they often took an anthropocentric approach—i.e., humans first—in matters such as culling animals as either a necessity or a precaution to protection human health [39].

The principle of “stewardship”—assigning the role of stewardship to humans in the protection and preservation of One Health—also raises concerns, such as whether it has a conflict-of-interest issue at heart. To a certain extent, sharing the planet Earth with animals and the wider eco-environment also means that, by default, humans are inevitably “competing” for a relatively finite pool of resources with residents of the floras and faunas, from securing foods and territory (e.g., the ruthless use of pesticides and herbicides as well as deforestation), leveraging available resources (e.g., industrialisation and domestication of animals), to eliminating potential risks (e.g., the culling of animals for fear of infectious disease outbreaks). Assigning humans as stewards of One Health is then similar to the impossible practice of asking players to be players, as well as judges, of the game simultaneously. The conflict of interest means that human stewards might be inherently biased to make fair decisions that would prioritise the interests and the well-being of animals over those of humans. Also, in light of the notable scarcity of research into interspecies communication, and the subjective conscious experience of animals [40–42], it is even questionable whether humans can have a true understanding of what constitutes animal well-being.

### One health *or* human-centred connected health?

These questions reflect one key issue that many One Health studies have yet to confront: in a world where humans’ health needs—let alone wants—have yet to be met, is it realistic to categorically treat the health of the animals—many of which have been domesticated as pets or a source of protein for humans, as an equal of the health of humans? Albeit seems ever-improving, it was not long ago when health professionals like doctors and nurses in highly medically advanced countries like the U.S. worked in COVID-19 wards with overused masks—if not maskless—or had to make-do with torn trash bags as protective “gowns” due to severe shortages of personal protective equipment (PPE) [43]. The coincided strain on medical supplies also meant that many doctors in affluent countries had to make the once unthinkable decision of denying life-saving medical care to patients due to a lack of beds, ventilators, or essential medicines [44, 45].

It is important to note that, in light of mundane yet ever-present threats  like food safety and security issues, antimicrobial resistance, and spillover infections [46–53], COVID-related threats to human health are but one of many. While our current society is often claimed as civil and modern, seemingly unstoppable massing killings in places like Gaza, for instance, have effectively created human-made hells where children and women, along with other vulnerable people, are starving to death, patients are dying from easily treatable wounds and curable illnesses due to prolonged absence of medical resources, that is, if hospitals can still function amid the attacks, while families and communities are either being decimated in droves or live in constant fear and other reverberation like grief, trauma, and beyond [54–56]. The increasingly immersive and 24/7 media coverage of these human sufferings means that the world at large—including people living in places far away from wars—can be subject and succumb to war-induced trauma [57]. In this “One World, One Hell”, where human sufferings have yet to be addressed, is it realistic to give equal prioritisation to animals in terms of resource allocation and beyond similar to those of humans? Are animals in yet-to-be-at-war societies really more precious than humans in war zones even in theory and principle?

While it can be argued that the blatant disregard for human lives and livelihoods of recent wars in Gaza and beyond make a mockery of almost everything public health holds dear, the scale, scope, and severity of human sufferings might be most difficult for One Health to confront. A rethink might be needed to recalibrate the underlying presumptions and principles of One Health, not least because, in addition to the abovementioned threats to global health, a pandemic caused by Disease X or Pathogen X—which could be more powerful and destructive than COVID-19—is hardly a low-probability threat [58–60]. For instance, rather than assigning equal weight to human health and animal health, perhaps it is more realistic to prioritize shared interests and common ground between humans, animals, and the overall environment, especially in times of grave global tension and geopolitical instability.

**Fig. 1:**
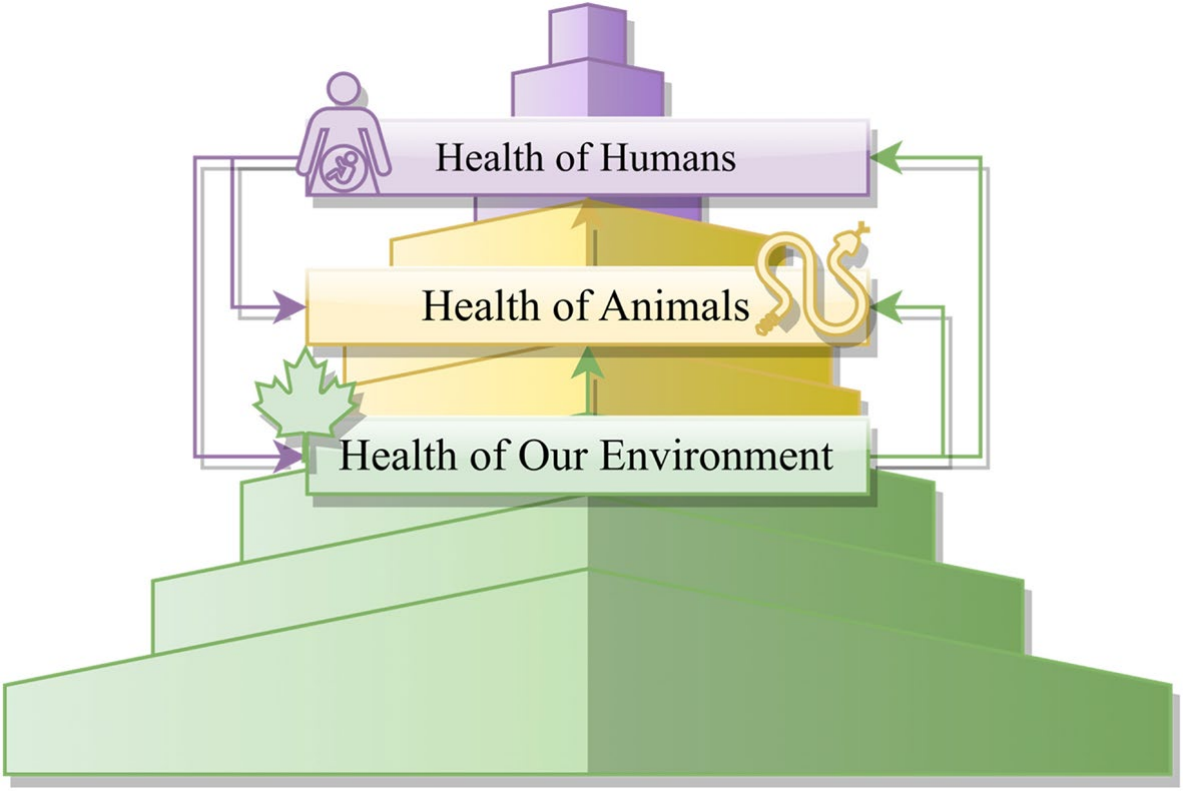
Visualisation of Human-Centred Connected Health

In other words, while the idea of treating all beings of the planet as equal is morally admirable and politically correct, in real-world practice, a clear and concrete commitment to human health while being actively considerate of the well-being of animals might have a greater chance of connecting the already deeply divided camps of society, including that of global health. The prioritisation of human health does not and should not dismiss the interconnectedness of human health, animal health, and environmental health. Rather, it creates a more tangible link between the health of different sectors (please see Fig. 1), while highlighting the instrumental role humans need to play in securing the safety and security of their living environment, from animal welfare (e.g., farming animals in an ecologically and ethically conscious manner) to global warming (e.g., actively pursuing sustainable and green consumption to mitigate global warming).

## Conclusion

Humans worked hard to make the current Anthropocene epoch a reality. Difficult—ethically controversial and morally uncomfortable—yet necessary clarifications need to be made across research fields to ensure the protection of human health is consistently prioritised across One Health fields. While compromises often seem to be impossible to tackle, global health leaders are and must be well-equipped to face the difficulties head-on. In a time when humans—healthy and ill, young and old, in war zones and beyond—face immense and ever-worsening health challenges on a daily basis, it would be a gross injustice to our own kind and humanity at large should we put political correctness over people’s welfare and well-being. To be united in health, we have to have the health needed to start—let alone finish—the job first. Human health has to be at the centre of One Health, in definition, in principle, and perhaps most importantly, in practice, clearly, consistently, and conclusively.

## Data Availability

Data are available upon reasonable request.

## Abbreviations

SARS: Severe acute respiratory syndrome
SARS-CoV-2: Severe acute respiratory syndrome coronavirus 2
COVID-19: Coronavirus 2019
OHHLEP: One Health High Level Expert Panel
U.S.: United States
